# *In vitro* abrasivity and chemical properties of charcoal-containing dentifrices

**DOI:** 10.1080/26415275.2020.1838284

**Published:** 2020-11-03

**Authors:** Foteini Machla, Aida Mulic, Ellen Bruzell, Håkon Valen, Ida Sofia Refsholt Stenhagen

**Affiliations:** Nordic Institute of Dental Materials, Sognsveien, Norway

**Keywords:** Charcoal, dentifrice, abrasivity

## Abstract

**Objective:**

Charcoal-containing dentifrices are gaining popularity, but scientific information on their effect on oral health is scarce. This study investigated properties of dentifrices that may affect dentine abrasivity, as well as their ability to adsorb fluoride, their pH and the presence of harmful substances.

**Materials and methods:**

The dentifrices NAO and COCO were subjected to the following analyses: abrasivity, expressed as mean abraded depth and relative dentin abrasivity (RDA), and surface roughness of extracted human molars (*n* = 30) after simulated brushing; fluoride adsorption measured as concentration change; pH measurements; detection of polycyclic aromatic hydrocarbons by gas chromatography–mass spectrometry. The products were compared to a reference dentifrice (Colgate^®^ MaxWhite), positive controls (ISO dentifrice slurry, activated charcoal for laboratory use) and a negative control (distilled water).

**Results:**

The mean abraded depths of NAO and COCO were not different (*p* > .05), but higher than the reference dentifrice and the negative control (*p* < .05). The RDA values of NAO, COCO and the ISO dentifrice slurry were higher than the reference dentifrice value (*p* < .05) by up to 10 times. The dentine surface roughness was higher after brushing with NAO, COCO and ISO dentifrice slurry compared to distilled water (*p* < .05). No change in mean adsorbed fluoride concentration was observed after 24 h (*p* > .05). Both NAO and COCO were alkaline (pH > 7). Analysis of NAO revealed the presence of naphthalene (112.8 ± 2.0 ng/mL).

**Conclusion:**

The charcoal-containing dentifrices were abrasive within acceptable limits set by ISO and did not adsorb fluoride. The presence of naphthalene in one product is a cause for concern.

## Introduction

The use of charcoal for oral hygiene purposes has long traditions in several countries [1]. Products containing charcoal are constantly being introduced to the market, advertising various advantageous properties, such as a whitening effect and detoxification [1].

Charcoal production is the result of the removal of water and other volatile constituents from carbon-based materials, such as bamboo, wood or coconut husk and shell. To increase the porosity, charcoal is activated upon exposure to high temperatures often in combination with gases during manufacturing [2,3].

To achieve physical removal of extrinsic stains dentifrices advertised as ‘whitening’ typically contain higher amounts of abrasives and detergents than conventional dentifrices [4]. No evidence pertaining to the claimed whitening and/or bleaching efficacy of charcoal dentifrices, without bleaching-promoting ingredients has been established [1,5].

The abrasive property of dentifrices may cause wear of the dental tissue itself [6–8]. Loss of tooth substance may cause dentine hypersensitivity [9], creation of gingival recession [6], abrasion on teeth [10] and affect tooth color [11]. In addition, change in surface roughness may affect the amount of biofilm formation and, thus, increase the risk of both caries and periodontal inflammation [12,13]. To evaluate tooth surface wear after brushing with dentifrices, both abrasivity, a quantitative measure of the amount of abraded material removed (mean abraded depth and relative dentin abrasivity (RDA)) and a qualitative measure, surface roughness, are commonly used [14]. Pertiwi et al. demonstrated that charcoal dentifrices increased the surface roughness of dental hard tissues, when compared to conventional dentifrices [15]. However, this observation may be product-specific.

Fluoride in oral hygiene products is recognized as protection against dental caries [16]. Activated charcoal is a universal adsorbent and has been shown to adsorb and remove fluoride from water and soil [17,18]. Fluoride may be added to charcoal-containing dentifrices by the manufacturer. However, further investigations are needed to assess how charcoal-containing dentifrices may interfere with the available fluoride in the oral environment. Fluoride adsorption could affect both fluoride added to the product or fluoride present in the oral environment from other sources.

Formation of polycyclic aromatic hydrocarbons (PAH) has been detected during the manufacturing of charcoals, such as from coconut shell, by pyrolysis [19], and the presence of PAHs has been quantified in waterpipe charcoal products among others [20–22]. Several PAHs are genotoxic and/or carcinogenic substances [23,24]. Despite the investigations into the presence of PAH in coconut shell charcoal, to the best of our knowledge, there are no investigations of chemical analyses of potentially harmful substances in charcoal-containing dentifrices.

Investigations into the physical and chemical properties of charcoal dentifrice use are limited [1]. Therefore, the aims of the present *in vitro* study were to investigate the dentine abrasivity and changes in surface roughness, adsorption of fluoride, dentifrice pH in water and artificial saliva, and the presence of harmful substances of two commercially available fluoride-free charcoal-containing dentifrices.

## Materials and methods

### Teeth

Thirty extracted caries-free, sound human third molars were cleaned from tissue remnants and stored in distilled water (DW) at 4 °C in a bio-bank (‘2013/413 NIOM tannbank’) approved by the Regional Committees for Medical and Health Research Ethics (REC, application no. 28748), Norway. The sample size was determined based on the relevant ISO standard [25].

### Dentifrices and controls

Two commercially available powdered charcoal dentifrices (Table 1), NAO Coco teeth whitening (Finally AS, Fyllingsdalen, Norway) (NAO) and COCO (SkinTechnologies AS Bergen, Norway) (COCO), were selected due to their advertising frequency. The dentifrices were obtained online and from cosmetic stores in Oslo, Norway. The commercially available dentifrice without charcoal, Colgate^®^ MaxWhite (Colgate-Palmolive Company, Lysaker, Norway) (MaxWhite) was included as a reference dentifrice for comparison (Table 1). The ISO dentifrice slurry [25] and DW were used to investigate dentine abrasivity as positive and negative control, respectively. To evaluate the fluoride adsorption by the charcoal dentifrices, an activated charcoal in powder form for laboratory use (C9157, Sigma-Aldrich, Oslo, Norway) acted as positive control.

**Table 1.** Ingredients and product forms of the investigated dentifrices according to manufacturers.

Dentifrice product name (abbreviation)	List of ingredients	Product form	Used in section
NAO Coco teeth whitening (NAO)	100% activated coconut shell charcoal	Powder	1–3
COCO (COCO)	organic activated coconut charcoal, orange seed oil, sodium bicarbonate, coconut oil, mint flavor	Powder	1–3
Colgate® MaxWhite white crystals (MaxWhite)	aqua, sorbitol, hydrated silica, glycerin, PEG-12, sodium lauryl sulfate, aroma, cellulose gum, tetrasodium pyrophosphate, cocamidopropyl betaine, sodium fluoride, sodium saccharin, hydroxypropyl methylcellulose, limonene, Cl 77891, Cl74160, Cl74260	Paste	1 and 3

### Dentine abrasivity

The abrasive effect of the charcoal dentifrices (NAO and COCO) was evaluated according to ISO 11609: 2010 with some modifications [25]. MaxWhite was used as a reference dentifrice.

#### Preparation of dentine specimens

Each tooth was sectioned at the cementoenamel junction using a cutting machine with a diamond blade (Accutom, Stuers, Ballerup, Denmark). To ensure the stabilization of the tooth, the buccal surface of the crown was slightly grinded with a water-cooled instrument (Knuth Rotor, Stuers). The crowns were embedded in epoxy resin (EpoFix, Stuers) in circular containers of specific dimensions, to fit in the sockets of the brushing machine and to protrude approximately 1 mm above the socket’s surface. A grinding machine (Pedemax, Stuers) was used to remove the enamel and to expose the outer part of the dentine of the crowns’ buccal surface. The specimens were polished using grinding papers (Silicon Carbide Paper P500-P1200, Stuers). Two pieces of adhesive tape were placed on the buccal dentine of the teeth parallel to each other to expose a section of dentine of approximately 1 mm width between the tape strips. The exposed buccal dentine section was parallel to the occlusal surface of the tooth. Following preparation, the specimens (*n* = 30) were randomly divided into five groups: NAO, COCO, MaxWhite (reference), ISO dentifrice slurry (positive control), and DW (negative control). A two-dimensional contact profilometer (Surftest SJ-201P, Mitutoyo, Scandinavia AB, Upplands Väsby, Sweden) was used to ensure initial dentine surface roughness (Ra) of ±0.3 μm. Ra is the mean absolute deviation of the peaks and valleys of the assessed profile from the mean line over a cut-off length and is the most commonly used roughness parameter [26]. The measurements were conducted on three random spots on the dentine, with a stylus tip radius of 5 μm, applying a force of 4 mN, a cut-off length of 0.25 mm and measuring speed of 0.25 mm/s. All specimens were stored in DW at 23 °C in between experiments.

#### Preparation of dentifrice slurries

The test slurries of the powders NAO, COCO, and the paste MaxWhite were prepared by adding 25 g of each dentifrice to 40 mL of DW, although this would result in higher final weight/volume ratios of particles in the charcoal dentifrices compared to MaxWhite. The ISO dentifrice slurry was prepared by adding 10 g of calcium pyrophosphate to 50 mL of the reference diluent (100 mL of glycerin, 5 g of carboxymethyl cellulose and 900 ml of DW and stirring slowly overnight at 23 °C [25]. Each slurry was prepared shortly before each brushing cycle and with vigorous stirring.

#### Brushing procedure

Simulated brushing was performed using a cross-brushing machine, in-house manufactured according to the instructions of the specification BS 5136:1981 [27]. Each tooth was brushed with 10 000 strokes and a weight of 150 g [25]. The direction of the brushing movement was parallel to the exposed length of dentine. The filaments (10 mm) of the brushes (Jordan, Orkla ASA, Oslo, Norway) were plane and soft. The bristles of the brush heads were positioned vertically to the surface of the specimen. One brush head was used for each group.

#### Abrasion and roughness measurements

To investigate the abrasivity and roughness of dentifrices on the tooth specimens, the corresponding profile of the exposed brushed zone was evaluated by an optical profilometer (S Neox, Sensofar, Terrassa, Spain) and interpreted with the SensoSCAN 6.3 software (Sensofar). A series of three stitched images (10% overlapping) were collected just outside and across the abraded zone, at three non-overlapping positions with an objective (EPI 20× v35) using the brightfield mode. The dimensions of the scanned areas were 2456 × 660 μm^2^ (3807 × 1023 pixels). These images were used to assess the RDA and Ra.

The mean of three measurements of the mean abraded depth of the exposed area was calculated for each group. The RDA value of each tested material was calculated according to the equation:

$$\text{RDA}=\;\left(A_{\text{mt}}\times 100\right){/A}_{\text{mr}}$$

as described in ISO 11609: 2010 (E), where *A*_mt_ is the mean abraded depth of the test dentifrice and *A*_mr_ is the mean abraded depth of the ISO dentifrice slurry [25].

The Ra parameter was measured inside and in the center of the brushed zone, vertical to the brushing direction (0.6 mm length). The mean of three not overlapping Ra measurements was calculated, applying a Gaussian filter of 0.08 mm to subtract the waviness of the profile.

### Fluoride adsorption

A solution of fluoride (10 mg/L) was prepared from a standard solution (100 mg/L F^-^, Thermo Scientific, Waltham, MA) using deionized water as solvent. Firstly, a baseline measurement of the fluoride concentration was conducted using an ion analyzer (ION 450, Meter Lab, Lyon, France) and a fluoride-selective electrode (Radiometer Analytical, Lyon, France). NAO, COCO and activated charcoal for laboratory use (positive control) was subsequently added (1 g/mL) and stirred at a speed of 150 RPM. Measurements were performed after 1, 3, 6, 12 and 24 h. During each measurement, a 5 ml aliquot was removed and filtered through a paper filter (White ribbon, 589^2^, Schleicher & Schuell, Dassel, Germany) and a syringe filter (0.45 µm, Millex, Millipore, Bedford, MA) to remove the charcoal powder from the sample solution. After adding an equal amount of total ionic strength adjustment buffer for fluoride (TISAB-F, Radiometer Analytical, Krefeld, Germany) to the filtered aliquot, the fluoride concentration was measured. Each aliquot was measured three times, the mean of which was calculated. Three experiments were performed.

### Chemical analyses

#### Gas chromatography-mass spectrometry analysis (GC-MS)

Suspensions of COCO in a mixture of methanol and toluene (6:1) and NAO in toluene (0.25 g/mL) were sonicated for 4 h at 45 °C. The choice of solvents was determined by initial qualitative screening studies. The suspension was centrifuged twice, first at 5000 RCF for 8 min (Heraeus Multifuge X3 FR, Thermo Fischer, Oslo, Norway) and secondly at 12,300 RCF for 5 min (VWR MicroStar 12, Leuven, Belgium) to remove the charcoal particles. Both qualitative and quantitative studies were carried out using gas chromatography mass spectrometry (GC-MS) analysis (Agilent, Santa Clara, CA). In the qualitative studies, an aliquot of the reaction solution (100 µL) was removed and added to acetone (400 µL). For quantification of PAHs, an aliquot (100 µL) was removed and added to a mixture of internal standard (phenyl methacrylate, 100 µL) and acetone (300 µL) prior to analysis. A standard mixture of the 16 PAHs (Sigma-Aldrich, Oslo, Norway) on the US Environmental Protection Agency’s (EPA) list and the instrument’s reference library of compounds (National Institute of Standards and Technology, Gaithersburg, MD) were used to identify hazardous PAHs for subsequent quantification. A calibration curve of known concentrations of the substance of interest was used for quantification. Three extraction experiments were carried out for each charcoal dentifrice.

Recovery experiments were performed to validate the extraction methodology. The limit of detection of naphthalene was 6 ng/mL. A solution of naphthalene in toluene (800 ng/mL) was prepared and extracted as described above. A standard curve of naphthalene was prepared to allow quantification. The percentage recovery was calculated based on the starting concentration of naphthalene and the amount quantified after extraction. Three extraction experiments were performed for the determination of recovery of naphthalene.

#### pH measurements

Solutions of NAO and COCO in both distilled water and artificial saliva were prepared (0.125 g/mL). The pH of MaxWhite (0.125 g/mL) in both solvents was measured as a reference. The artificial saliva contained (1/L) CaCl_2_·2H_2_O (0.7 mmol), MgCl_2_·6H_2_O (0.2 mmol), KH_2_PO_4_ (4.0 mmol), KCl (30.0 mmol) and 4-(2-hydroxyethyl)-1-piperazineethanesulfonic acid (HEPES) buffer (20.0 mmol) [28]. The pH was corrected to 7 by the addition of 1 mol/l sodium hydroxide. After stirring at 1000 rpm for 45 min at 23 ± 2 °C, the pH of the solutions was measured using a pH meter (Sension+, Hach, Loveland, CO). Three solutions of each group were measured. The mean pH values ± SD were calculated.

### Statistical analyses

The statistical analysis was performed by SPSS Statistics software, version 19 (IBM Corp., New York, NY) and the level of significance was set at *p* < .05.The mean abraded depth and surface roughness (Ra) were analyzed using the Kruskal–Wallis test followed by the Dunn’s multiple comparison test, as the assumption of equal variance was not met. To analyze results from the fluoride adsorption study, one-way ANOVA was performed at time point 0 and 24 h. The pH values were analyzed using one-way ANOVA followed by the Tukey’s post-hoc test.

## Results

### Dentine abrasivity

**Figure 1.**
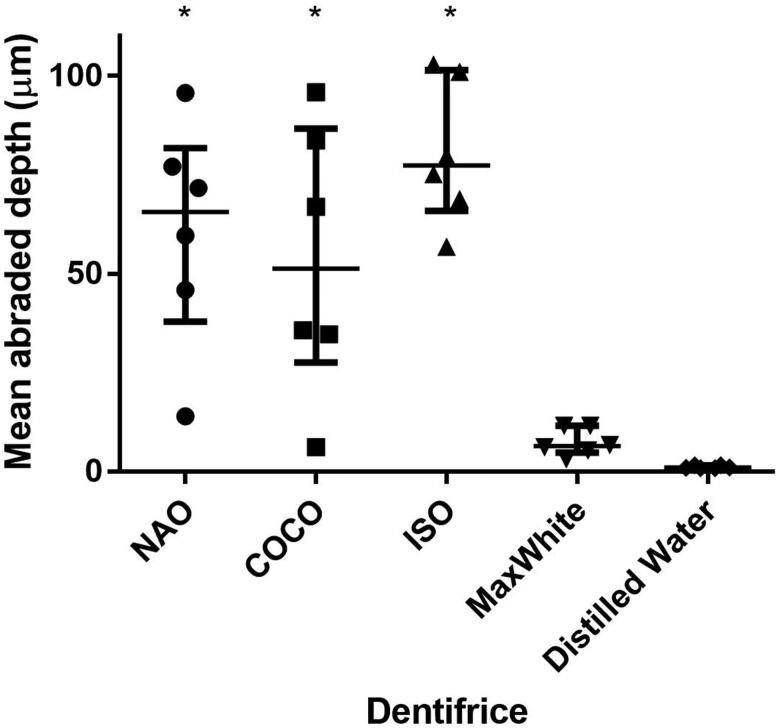
The mean abraded depths (µm) of the tested dentifrices, reference and controls displayed as median and interquartile range. *Significantly different from DW, *p* < .05 (*n* = 6).

**Figure 2.**
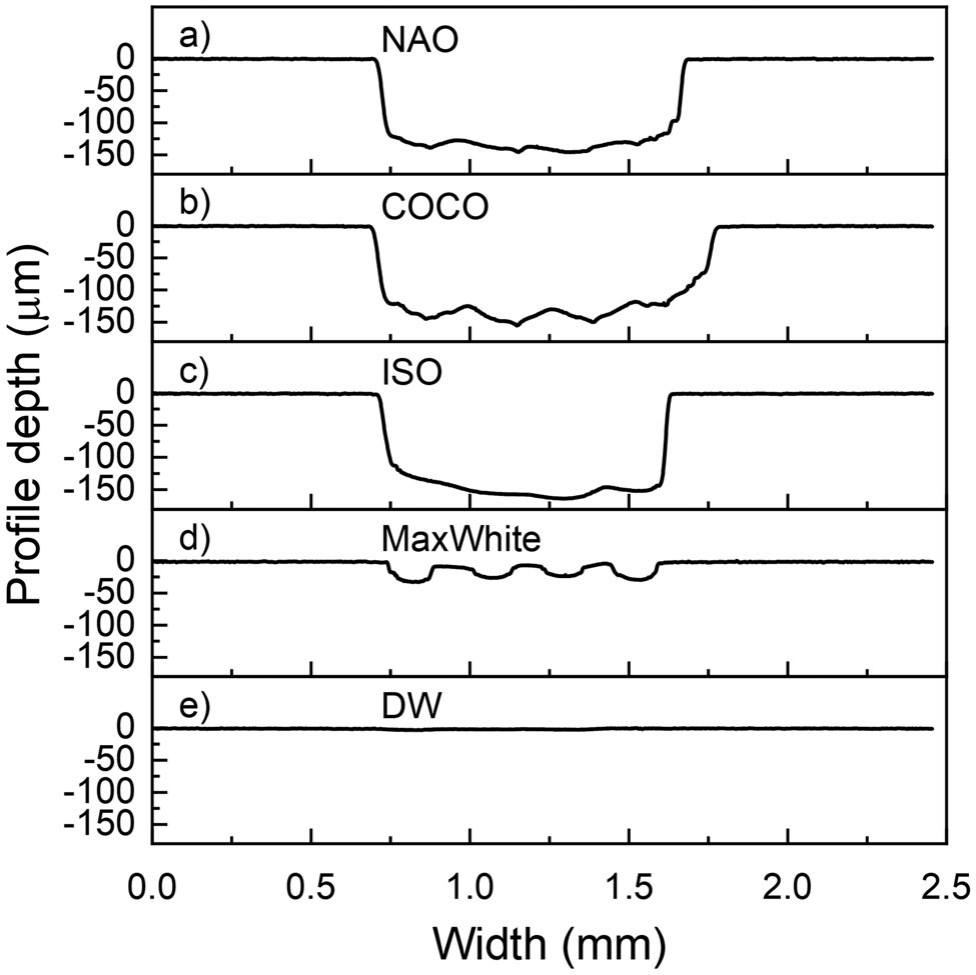
Illustration of the brushed zone depth profile widthwise of the tooth specimen. One typical tooth specimen from each group (a)–(e) is shown.

Brushing with NAO, COCO and ISO dentifrice slurry resulted in larger mean abraded depths compared to brushing with DW (*p* < .05) (Figure 1). The mean depth of the brushed zone varied along the evaluation length (Figure 2).

The RDA values (not adjusted for the difference in weight to volume of particles) of NAO, COCO and ISO dentifrice slurry were 75, 66 and 100, respectively and up to about 10 times higher than that of MaxWhite (RDA = 9).

**Figure 3.**
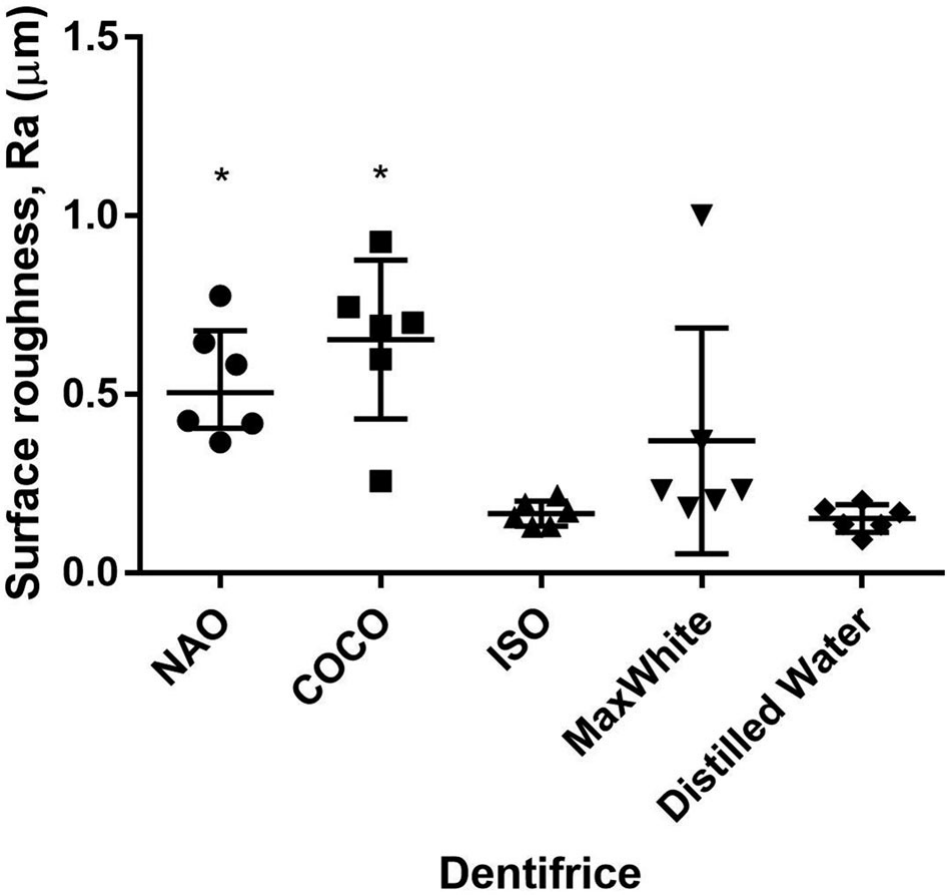
The surface roughness (Ra) in μm of the investigated dentifrices, reference and controls displayed as median and interquartile range.*Significantly different from ISO dentifrice slurry and DW, *p* < .05 (*n* = 6).

Brushing the specimens with NAO and COCO resulted in higher Ra values than those of controls (*p* < .05). The Ra values of NAO, COCO and MaxWhite were not different (*p* > .05) (Figure 3).

### Fluoride adsorption

**Figure 4.**
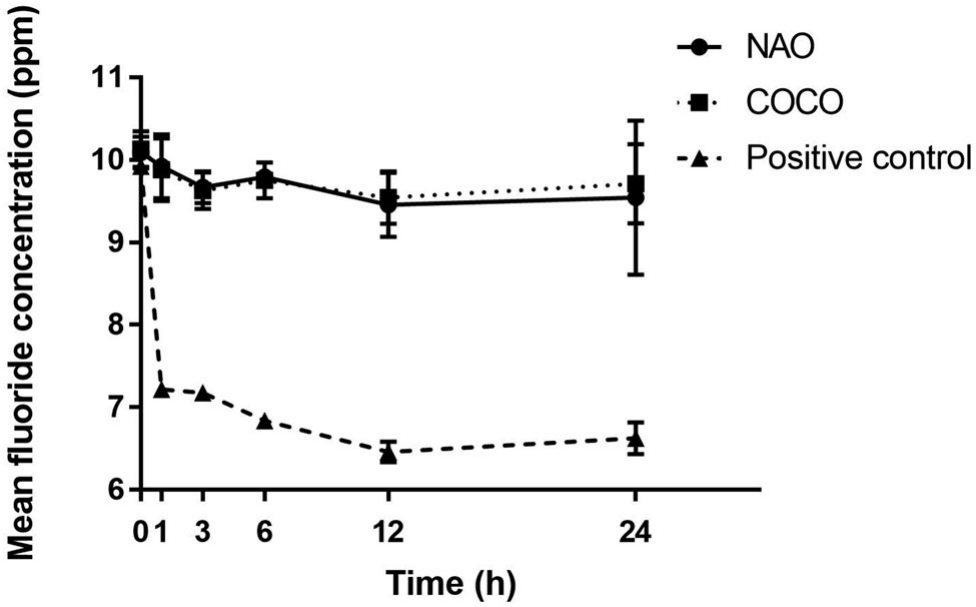
Mean fluoride concentration (ppm ± SD) of the investigated dentifrices detected between 0 and 24 h (*n* = 3).

No reduction in mean fluoride concentration in NAO and COCO was observed after 24 h (*p* > .05) while that of the charcoal positive control was reduced (*p* < .05) (Figure 4).

### Chemical analyses

Qualitative analysis of COCO revealed the presence of menthol, menthone, neoisomenthol, limonene, eucalyptol and vanillin. Compared with the standard PAH mixture, the chromatogram of NAO showed a peak corresponding to naphthalene. Quantitative analysis gave naphthalene in an amount of 112.8 ± 2.0 ng/mL. The naphthalene recovery was 99% following extraction.

The pH of NAO, when dissolved in either DW or artificial saliva, was higher than both COCO and MaxWhite (*p* < .05) (Table 2).

**Table 2.** pH ± SD of the investigated dentifrices in distilled water and artificial saliva.

	NAO	COCO	MaxWhite
Distilled water	10.2* ± 0.1	8.4 ± 0.7	8.1 ± 0.1
Artificial saliva	9.0* ± 0.1	7.7 ± 0.0	7.1 ± 0.0

*Significantly different from COCO and MaxWhite (*p* < .05) (*n* = 3).

## Discussion

Based on the RDA results of the current investigation, both fluoride-free charcoal-containing dentifrices were classified as medium abrasive (RDA 66-100). The size, size distribution, shape, hardness of the charcoal particles together with applied load will influence the abrasivity of the dentifrice [29]. The abrasivity is shown to increase with particle size up to the point where the particles escape the bristles and are moved aside by the toothbrush. The relatively large variation observed in mean abraded depth between the charcoal-containing dentifrices and control groups, may reflect the distribution of the particle size, and composition as well as differences in the dentine of the individual molars [30]. The substance loss and roughness caused by NAO and COCO in the present study confirm similar results reported on charcoal dentifrices [15]. The effect of abrasivity of a dentifrice on tooth surfaces should be assessed through both RDA and Ra measurements to obtain information on both the amount of tooth substance abraded and the roughness of the resulting surface [14]. Comparison between dentifrices in different formulations is challenging. Even though the weight of abrasive material per volume in the ISO dentifrice slurry is lower than in the charcoal dentifrices, the latter gave a higher RDA value. ISO recommends that dentifrices have an average RDA between 60 and 100 [25], and MaxWhite showed an average RDA of 9. It must be noted that the charcoal dentifrices are in powder form, while MaxWhite is a paste with several ingredients. All investigated dentifrices were diluted with distilled water, thereby, the obtained relative concentration of particles were lower for MaxWhite. A limitation of the study is that the particle size, distribution, composition and morphology vary between the tested dentifrices. Since only two charcoal-containing dentifrices were tested in the current study, the abrasivity results cannot be generalized to all such dentifrices, especially the ones that are in the form of a paste.

Conventional dentifrices are a daily source of fluoride with the intention to promote the remineralization of enamel and dentine, and thereby protect against dental caries [16]. Thus, the ability of a dentifrice to adsorb fluoride would be a disadvantage. The charcoal for laboratory use (positive control) did adsorb fluoride in contrast with the two tested charcoal-containing dentifrices. This suggests that the two charcoal-containing dentifrices investigated may not interfere with fluoride availability in the oral environment.

Although the pH values of the dentifrices in the present study were reduced by the buffering capacity of artificial saliva, the pH value of NAO remained higher than those of COCO, fluoridated whitening [31] (pH 4.22–8.35) and commercial fluoridated dentifrices [32] (pH 7.0). It is uncertain how the alkaline pH of the charcoal dentifrices affects the oral tissues [33], but any effect is likely to be a function of time and frequency of the application.

The presence of naphthalene in NAO is unsurprising as naphthalene is reported as the most abundant PAH in biochar (product of pyrolysis used for agricultural and environmental applications rather than fuel) [34]. According to European Union regulation, the presence of naphthalene in cosmetic products is prohibited [35]. The toxicity of naphthalene is outlined in a report by the National Toxicology Program [36]. A limitation of the present study was that the presence of PAHs only was investigated in these charcoal-containing dentifrices. An alternative extraction protocol (Soxhlet extraction) or the use of liquid chromatography–mass spectrometry (LC-MS) rather than GC-MS may have revealed other PAHs [37]. LC-MS is normally capable of achieving lower limits of detection compared to GC-MS, depending on the molecular weight of the compound of interest. Elemental analysis of coconut-based charcoals used in waterpipes showed larger amounts of heavy metals (lead, zinc, iron, cadmium, aluminum, cobalt and chromium) compared to the content of cigarettes, therefore, the presence of other hazardous substances cannot be disregarded [38]. Furthermore, only one batch of each dentifrice was used and the country of origin of the charcoal was unknown. Thus, it is also reasonable to expect variation between batches or types of charcoal-based dentifrices [39].

According to Brooks et al. who investigated advertising claims of fifty charcoal products, there are ‘insufficient clinical and laboratory data to substantiate the safety and efficacy claims of charcoal and charcoal-containing dentifrices’ [1]. Of the products investigated, among other characteristics, 46% advertised detoxification, 30% claimed to remineralize, strengthen or fortify the teeth, 28% to be low abrasive or gentle to the enamel and 8% to contain fluoride [1]. Even though the current study investigated some properties of charcoal dentifrices, further research should be implemented to investigate other advertised features, such as their degree of biocompatibility, their antimicrobial effect, and bleaching and/or whitening effect [1].

## Conclusion

The tested charcoal-containing dentifrices were abrasive within acceptable limits set by ISO and did not adsorb fluoride. One of the tested products (NAO Coco teeth whitening) contained naphthalene, which is in breach of EU regulations. The role of charcoal-containing dentifrices in promoting oral and dental health is questioned.
